# Metal‐Free Custom‐Made Zirconia Implants—A Prospective 5‐Year Follow‐Up Single‐Arm Clinical Trial

**DOI:** 10.1111/cid.13404

**Published:** 2024-11-06

**Authors:** Jantien H. W. de Beus, Marco S. Cune, Henny J. A. Meijer, Gerry M. Raghoebar, Ulf Schepke

**Affiliations:** ^1^ Department of Periodontology University of Groningen, University Medical Center Groningen, Center for Dentistry and Oral Hygiene Groningen Netherlands; ^2^ Department of Oral Maxillofacial Surgery University of Groningen, University Medical Center Groningen Groningen Netherlands; ^3^ Department of Restorative Dentistry University of Groningen, University Medical Center Groningen, Center for Dentistry and Oral Hygiene Groningen Netherlands; ^4^ St. Antonius Hospital, Department of Oral Maxillofacial Surgery Prosthodontics and Special Dental Care Nieuwegein Netherlands

**Keywords:** dental implants, metal‐free dentistry, zirconia

## Abstract

**Background:**

Dental implants made of zirconia (ZrO_2_) are a potential alternative for titanium implants in dentistry because of their good biocompatibility, mechanical properties and excellent aesthetic results. However, solid long‐term scientific data to prove clinical success of ZrO_2_ implants are scarce.

**Aim:**

The aim of this study was to describe and to examine the clinical performance of custom‐made two‐piece ZrO_2_ implants, to identify possible influencing factors: a) manipulation of the implant after placement and b) the occlusal scheme on the survival rate, and to evaluate the performance of the implant‐supported crown. This follow‐up study collected and examined the 5‐year data to answer the main question: What are the survival and the success rates of custom‐made ZrO_2_ implants in the maxillary premolar region after 5 years?

**Material and Methods:**

Of the 31 included patients in this prospective 5‐year follow‐up single‐arm clinical trial, 30 received a custom‐made ZrO_2_ implant to replace a missing single maxillary premolar, which was subsequently restored with a lithium disilicate crown. Parameters regarding clinical performance, marginal bone‐level (MBL) changes, and patient‐related outcome measures (PROMs) were assessed preoperatively, at the baseline, as well as 1 and 5 years after crown placement. Chances of survival and success of the implant were calculated and displayed using Kaplan–Meier statistics. Kaplan–Meier survival analysis was also performed with stratification based on the variables “manipulation of the implant prior to impression taking” and “occlusal scheme” and compared using log‐rank tests. Bone‐level moderation in time was compared using a paired samples *t*‐test. Patient's expectations and satisfaction after 5 years were compared as a measure of fulfilled expectations, using a Wilcoxon signed‐rank test. Performance of the implant‐supported crowns was evaluated using validated criteria.

**Results:**

Survival and success probabilities after 5 years were, respectively, 75.8% (95% CI [60.0%; 91.0%]) and 71.0% (95% CI [54.0%; 88.0%]) for the custom‐made ZV3 implants. No significant differences in survival rate were found after stratification on “manipulation of the implant” and on “occlusal scheme.” Mean bone‐level alteration between baseline and the first follow‐up was +0.06 mm (95% CI [−0.23 mm; 0.12 mm]; SD = 0.42 mm) and between baseline and the second follow‐up was +0.04 mm (95% CI [−0.35 mm; 0.26 mm]; SD = 0.54 mm). Patients' satisfaction for patients with implants still in function after 5 years was 91.7% (IQR = [90.5%–97.3%]), indicating satisfaction with the treatment. Pooled satisfaction in patients with successful implants after 5 years was significantly higher than patients' expressed expectations before treatment. None of the crowns failed, and no interventions were required.

**Conclusion and Clinical Implications:**

Survival rate of these particular ZV3 implants in our study was lower than expected and clinically not acceptable. Hence, ZV3 implant placement as applied in this study cannot be recommended for clinical practice. Further research on the different appearances of mechanical failure in ZrO_2_ implants would be highly recommended before a larger prospective randomized clinical trial is conducted to evaluate treatment with custom‐made ZrO_2_ dental implants.

## 1 Introduction

Dental implants have offered a reliable solution for the replacement of missing teeth [1]. A multitude of base materials was used for dental implants throughout the years [2]. Ideal material properties for dental implants should include adequate toughness, strength, corrosion, wear, fracture resistance, and biocompatibility [3].

Until now, titanium and its alloys are seen as the “gold standard” material for dental implants given their good mechanical and biological properties and a multitude of positive long‐term survival rates in different indications are documented [4, 5, 6].

However, adverse effects attributed to titanium or titanium alloys [7, 8, 9], such as a higher risk of undesirable allergic reactions, cellular sensitization [10, 11], and a clinically visible gray hue of the peri‐implant tissues [12, 13, 14], trigger the demand for an alternative dental implant material, with even better biocompatible and aesthetic properties [15].

High strength ceramics, especially yttria‐stabilized tetragonal zirconia polycrystal, might be a promising alternative to titanium [16, 17, 18]. Implants made of zirconia (ZrO_2_) are biocompatible [19, 20] and have good mechanical properties that make them suitable for clinical use [21]. ZrO_2_ is a strong and tough but delicate material, and therefore, it should be handled with care, where mechanical preparation after sintering could impair the material's strength [22], and the actual mechanical strength of the implant can depend on the design of the implant and the manufacturing process [23]. ZrO_2_ shows comparable results concerning bacterial colonization and tissue health to titanium [24]. Additionally, the ZrO_2_ implants have an acceptable white color and return excellent aesthetic results [25]. Taken together, these results suggest that ZrO_2_ might meet the requirements of an alternative implant material to titanium, assuming that the clinical performance is comparable.

Despite these findings about the role of ZrO_2_ dental implants in dentistry, literature still does not provide consensus on whether ZrO_2_ is a viable option. Pieralli et al. [16] conducted a systematic review, concluding that 1 year survival and success rates of ZrO_2_ dental implants are both promising and comparable to data of titanium implants [16]. However, more high‐evidence studies are needed to confirm the long‐term predictability of ZrO_2_ implants in terms of success and survival [17], especially when it comes to two‐piece implant designs [26]. Hashim et al. [27] included a multitude of indications and different types of ZrO_2_ implants to help the general dental practitioner with the choice for ZrO_2_ as a dental implant material in clinical practice [27]. However, the main conclusion of this systematic review was that there are no clear indications for ZrO_2_ implants so far and pointed out the nescience regarding long‐term outcomes and specific kinds of failure.

The studies reviewed so far lack adequate focus on the intercomparability of the research. Furthermore, long‐term clinical performance studies are needed to validate the outcome of ZrO_2_ as a material for dental implants [27, 28, 29] since implants are expected to be a long‐term solution for single‐tooth replacement in terms of success, survival, and improvement of the patient's oral health related quality‐of‐life (OHQoL) [1, 30, 31, 32]. Solid long‐term scientific proof of clinical success of ZrO_2_ implants is scarce, although several short‐term clinical performance studies have been conducted [28, 33, 34, 35, 36, 37]. Adverse events need to be monitored to find a clear indication for the clinical use of ZrO_2_ as a material for dental implants in terms of success and survival.

The aim of this study was to describe and examine the clinical performance of custom‐made ZrO_2_ two‐piece dental implants, particularly ZV3 implants, and to evaluate its implant‐supported fixed crown. Possible influencing factors, manipulation of the implant after placement and the occlusal scheme on the success and survival rate, were evaluated to get a better understanding of the indications for this type of implant.

## 2 Materials and Methods

### 2.1 Study Design and Patients

The present study was designed as a prospective single‐arm clinical trial with results up to 6 years. Consecutive patients missing a single maxillary premolar with an indication for replacement by means of an implant were candidates for inclusion. In‐ and exclusion criteria were adopted from a previous study [38] and are presented in Table 1. Patients who met the criteria were provided with written and verbal information about the study and provided informed consent. Permission from the medical ethics committee of the University Medical Center Groningen (UMCG), the Netherlands, was granted (METc number 2013/048, ABR number NL 43024.042.13).

**TABLE 1:** Inclusion and exclusion criteria.

Inclusion criteria
Patient is at least 18 years old
Missing first or second premolar in the maxilla
Wish to replace the missing premolar with an implant
Willing to sign the informed consent
Bone height ≥ 10 mm beneath the maxillary sinus
Exclusion criteria
Missing teeth mesial or distal from implantation site
Bone augmentation needed prior to implant placement
Orthodontic treatment at the time of impression taking
Severe bruxism
Acute periodontitis
History of implant loss
Documented extreme gagging reflex
Poor medical condition (ASA^a^ score three or higher)
Previous therapeutic radiation of the head–neck region
Chronic pain in orofacial system
Younger than 18 years at time of inclusion
Reduced mental capacity

^a^ American Society of Anesthesiologists.

**FIGURE 1:**
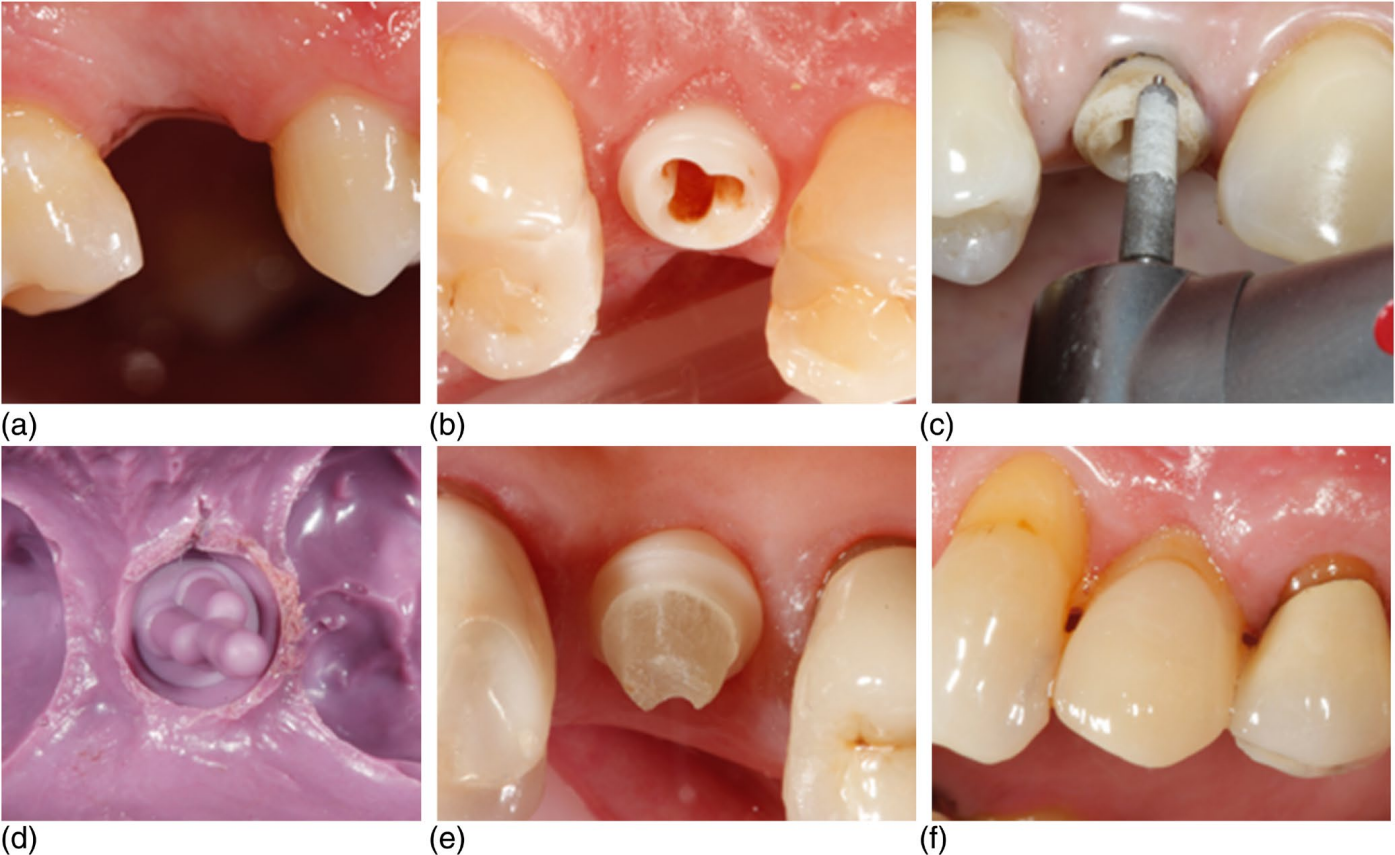
Workflow of the implant treatment (a) missing premolar, (b) implanted with a ZV3 implant, if needed (c) manipulation of the implant with a diamond burr (water cooling not in the picture), (d) impression with a polyether impression material, (e) ZV3 implant with an adhesively cemented original glass fiber post, and (f) ZV3 implant with an adhesively cemented lithiumdisilicate crown.

The population for this study included 31 patients, 30 of whom received a custom‐made ZrO_2_ implant at the place of a missing single maxillary premolar (Figure 1a). One patient was excluded from the study due to a protocol violation at implant placement, and another type of implant instead of the allocated type was placed. Primary outcomes were survival and success of the implant and of the implant‐supported crown.

### 2.2 Implants and Treatment Protocol

The ZV3 individual implants (ZV3 individual implants, Zircon Vision GmbH, Wolfratshausen, Germany) were designed on the basis of the available local bone volume measured on a CBCT and an intraoral scan, as also described by Brüll et al. [39]. The minimal required bone volume surrounding the implants was 1 mm, resulting in a variety of implant lengths and neck designs. After designing, the implants were milled from isostatically pressed, yttria‐stabilized and cerium co‐stabilized ZrO_2_ (MKM Engineering). After milling, the implants were air particle abraded prior to sintering.

Implant placement was performed after a minimum healing period of 3 months after extraction. One hour preceding the surgical intervention, patients received antibiotic prophylaxis (2 g amoxicillin or 600 mg clindamycin in case of penicillin allergy, taken orally). One day before surgery, patients began using oral disinfection (0.12% chlorhexidine mouthwash) twice daily and for a period of 10 days post surgery. All surgical interventions were performed under local anesthesia. Thirty patients received an individually designed ZrO_2_ implant from renowned operators with broad experience in titanium implant placement.

Accordingly, the implants in this study were placed in the native bone rather than performing extended bone augmentation, in cases where enough palatal bone was present to establish primary stability, as titanium implant placement would have been performed in our clinic.

After a healing period of 12 weeks (Figure 1b), restorative treatment commenced. If necessary, the implant was manipulated intraorally where needed (buccally) with a diamond burr with sufficient water cooling (Figure 1c) for aesthetic purposes before making the impression with a polyether (Figure 1d) (Impregum, 3 M ESPE, Seefeld, Germany) in a closed semi‐individual impression tray (Border‐Lock, Clan Dental, Maarheeze, the Netherlands). Subsequently, the lithium disilicate crowns (IPS e.max Press, Ivoclar Vivadent, Schaan, Liechtenstein) on original ZV3 fiberglass posts (Figure 1e) were manufactured. Both were cemented adhesively (RelyX Ultimate, 3 M ESPE, Seefeld, Germany) simultaneously (Figure 1f), approximately 15 weeks after implant placement. Excess cement was removed under inspection with a microscope. All implant crowns were manufactured by the same dental technician at a single dental laboratory (Elysee Dental Labs, Groningen, The Netherlands). Following the placement of the crown, occlusion was established with light contacts in maximum occlusion and a wide freedom in centric relation [40, 41]. Static and dynamic occlusions were checked with articulation paper (Artikulationspapier, Bausch Arti‐Check, Cologne, Germany), and comprehensive oral hygiene instructions were delivered. The restorative treatment protocol was administered by a sole, proficient operator (US).

### 2.3 Data Collection and Follow‐Up

Various parameters related to the implant and adjacent teeth were evaluated at four different time points: prior to implant placement (T0), 2 weeks after crown placement following implant placement (T1) as a baseline, 1 year after crown placement (T12), and approximately 5 years after baseline measurements (T60). Patients were invited for follow‐up consultations if the implant was still in situ. All parameters were assessed by a single researcher at all times. The primary focus of the study was to determine survival of the ZV3 implant. The secondary focus was a) to describe the success of the implant‐crown complex by the assessment of several parameters regarding clinical performance, MBL change, PROMs and b) to determine possible influencing factors on implant survival such as manipulation and the occlusal scheme and c) the performance of the implant‐supported crown.

#### 2.3.1 Implant Survival

Implant survival was defined as the implant being functional in situ. An implant had failed when it failed to osseointegrate (early failure), was lost due to peri‐implantitis (late failure), or in case of mechanical failure, such as implant fracture [18].

To determine possible influencing factors on implant survival, the original cast models of all subjects were studied retrospectively to determine the kind of occlusal scheme during dynamic occlusion and whether the implant had been manipulated buccally prior to impression taking. The occlusal scheme could be either canine protection or group function.

#### 2.3.2 Implant Success and Performance of the Implant‐Supported Crown

Success criteria included the absence of implant mobility, radiographic peri‐implant radiolucencies, vertical MBL of loss more than 0.2 mm annually after the first year of implant service, pain, and inflammation of the peri‐implant tissues or nerve damage [42].

Standardized intraoral radiographs were obtained at T1, T12, and T60 to determine possible peri‐implant radiolucencies and MBL change, using a custom‐made x‐ray holder to ensure equal projection. MBL was measured using computer software (DicomWorks, Biomedical Engineering, University Medical Center Groningen, the Netherlands) as described by Guljé et al. [43]. Distances from the distal and mesial shoulder of the implant to the first marginal bone on the mesial and distal sites of the implant were measured by two researchers independently. The intersection width of the top of each of the individually designed implants was taken as a reference point for the measurements. Values were averaged between measurements of both researchers. If values between measurements differed more than 0.40 mm, the researchers sought consensus on a third measurement. For each implant the most severe MBL change was taken. Modifications in MBL change were set by these most severe values of T1 and T12 and T1 and T60. Deposition of marginal bone was indicated by a positive value. Also, the average of the mesial and distal MBL was calculated to determine changes over time.

Clinical parameters consist of the presence of plaque [44], presence or absence of dental calculus, probing depth in millimeters, and bleeding score [44]. They were measured at the mesio, medio‐, and distobuccal sides of the implant and neighboring teeth using a calibrated probing force of 0.25 N and a standardized probe (Hawe Clickprobe, Hawe Neos Dental, Bioggio Switzerland), and mean probing depths per implant were included in data processing and analysis. Inflammation of the peri‐implant tissues was determined according to the consensus reached at the consensus meetings VI and VII of the European Workshop on Periodontology [45, 46] as also described by Slot et al. [47]. Peri‐implant mucositis was described when MBL < 2 mm and peri‐implantitis when MBL > 2 mm, both in combination with bleeding on probing and/or suppuration.

An implant was considered as a failure in terms of implant success if one or more of these criteria were met. Furthermore, during follow‐up, the implant was examined clinically for possible mobility, pain, and nerve damage.

Additionally, the evaluation of the implant‐supported crown was described with the help of the FDI World Dental Federation clinical criteria [48]. The criteria consisted of aesthetic, biological, and functional properties, each with subcategories. Complications were described if intervention was required. When an implant was lost, this was considered to have no impact on the success or survival rate of the crown.

#### 2.3.3 Patient‐Reported Outcomes

PROMs were measured by a questionnaire containing 16 statements regarding the patient's emotional, functional, aesthetic, and overall satisfaction with the restoration. The questionnaire was adapted from the questionnaire for patient's satisfaction of Guljé et al. [43]. The answers of the visual analog scale (VAS) that was used were calculated to percentages ranging from complete disagreement, calculated to 0%, to complete agreement, calculated to 100%.

### 2.4 Statistical Analysis

The Research Electronic Data Capture system (REDCap) was utilized for data management. Descriptive statistics were employed to report variable characteristics (e.g., distribution) prior to testing. Normality assumptions were evaluated through graphical representations of the data. If visual examination of the normality assumptions was inconclusive, a Kolmogorov–Smirnov test was applied to check for normality. All analyses were performed using a statistical program (SPSS, version 28.0 for Mac, SPSS inc, Chicago, USA), with significance set at *p* < 0.05. To compute survival graphs and 95% CI, a separate software was used (MedCalc Statistical Software version 20.0.4, Ostend, Belgium; 2021).

Implant survival and success rates were calculated using Kaplan–Meier statistics. Confidence intervals of 95% were given over the results at 5 years.

To determine the influence of the manipulation of the buccal edge of the implant and the occlusal scheme of the subject on the survival of the ZV3 implant, the Kaplan–Meier survival analysis was also performed with stratification by means of the defined groups, and a log‐rank test was performed.

To determine possible differences in average MBL, this was compared over time (baseline, T12 and T60), and a repeated measures ANOVA with a post hoc Bonferroni test was conducted. Patients' expectations and satisfaction at second follow‐up were compared across time using Wilcoxon signed‐rank tests.

## 3 Results

### 3.1 Research Population

The basic demographic and clinical data of the research are presented in Table 2. Mean age of the participants was 52.5 years (range 23–75 years) at the time of the implant placement. The average follow‐up survival time was 48.3 months (range 1–77 months).

**TABLE 2:** Basic demographic and clinical data of the research population that was treated with an ZV3 zirconia implant (*n* = 30).

Gender	Male (*N* (%))	15 (50)

Female (*N* (%))	15 (50)
Mean age^a^	Mean (range)	52.5 (23–75)
Tooth	14 (*N* (%))	8 (26.7)
	15 (*N* (%))	8 (26.7)
	24 (*N* (%))	5 (16.7)
	25 (*N* (%))	9 (30)
Types of occlusal scheme	Canine guidance (*N* (%))	10 (33.3)
	Group function (*N* (%))	20 (66.7)
Implant manipulated	Manipulated (*N* (%))	14 (46.7)
	Not manipulated (*N* (%))	16 (53.3)

^a^ At the time of implant placement.

**FIGURE 2:**
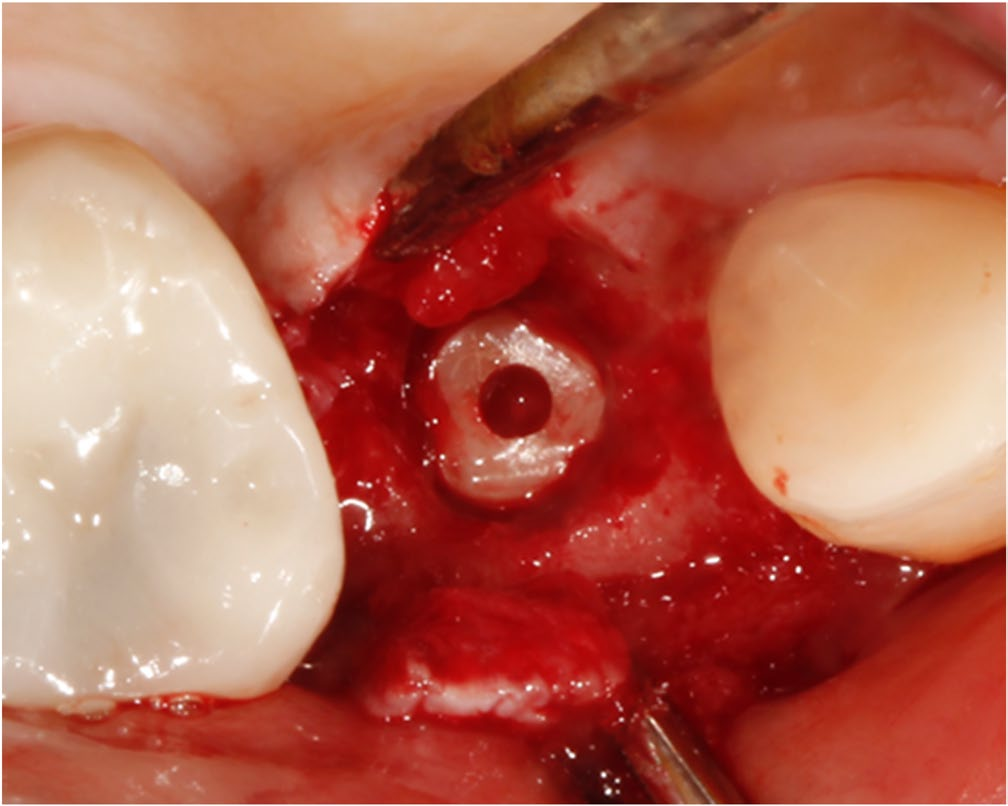
Apical intraosseous part of a horizontally fractured implant in situ. Note the to some extent exentric (palatal) placement of the implant.

**FIGURE 3:**
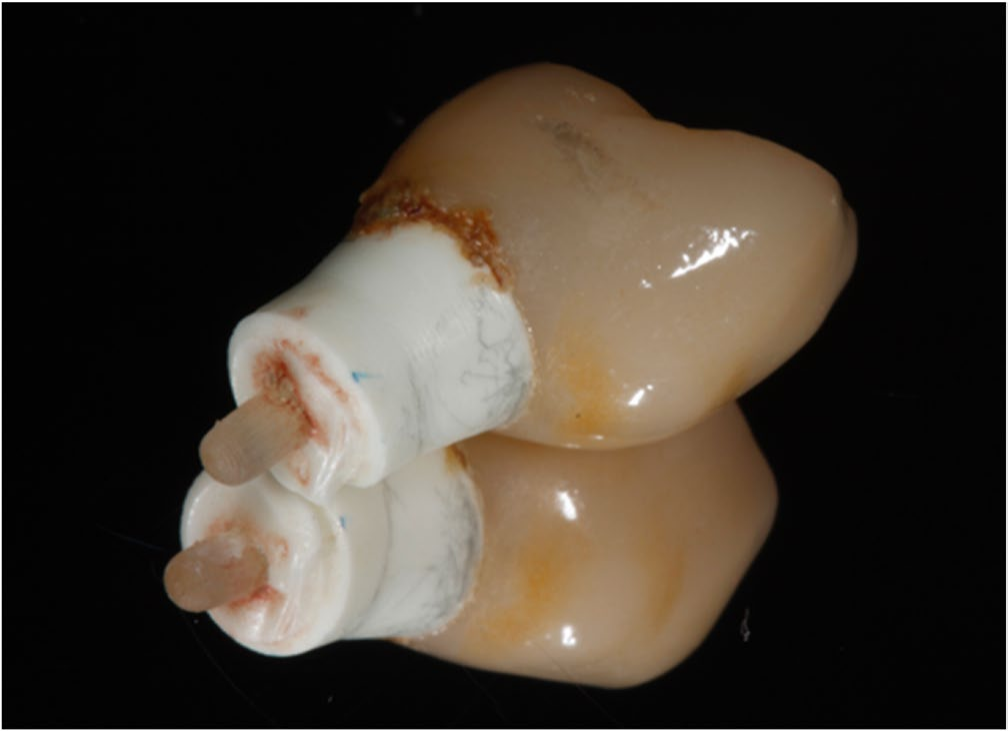
Upper fragment of horizontally fractured implant with the cemented implant‐supported crown.

**FIGURE 4:**
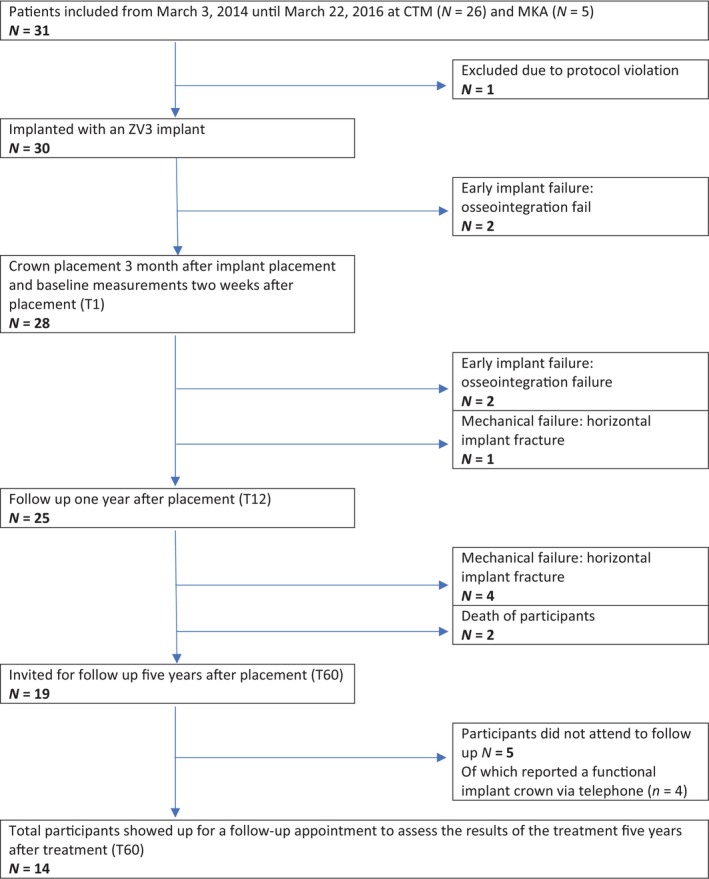
Consort flow diagram. Treatment consisted of implantation with a zirconia ZV3 implant. Patients were included for participation in this study at the Center for Dentistry and Oral Hygiene, Department of Restorative Dentistry, and the Department of Oral and Maxillofacial Surgery (University of Groningen, University Medical Hospital, Netherlands). After a healing period of 12 weeks, a lithium disilicate crown was manufactured and cemented. Several parameters were assessed at preoperative state (T0), the baseline 2 weeks after placement of the crown on the implant (T1), 1 year after placement at first follow‐up (T12), and 5 years after placement at second follow‐up (T60).

Four out of 30 patients experienced early implant failure (*n* = 4) due to failed osseointegration. For two patients with failed osseointegration, the lithium disilicate crown had already been manufactured and cemented. Five patients experienced mechanical failure (*n* = 5) due to horizontal implant fracture (Figures 2 and 3). Two patients died during the course of the study, while the implant was still in situ (*n* = 2). For this 5‐year follow‐up, 19 patients received a recall appointment as their implant was still in situ. Five patients did not attend (*n* = 5). Of those five patients, four implant‐supported crowns were reported telephonically by the participant to be in function without any problems. One participant could not be reached. Eventually, 14 patients participated in the follow‐up appointment approximately 5 years after treatment, and a consort flow diagram is provided in Figure 4.

### 3.2 Implant Survival

**FIGURE 5:**
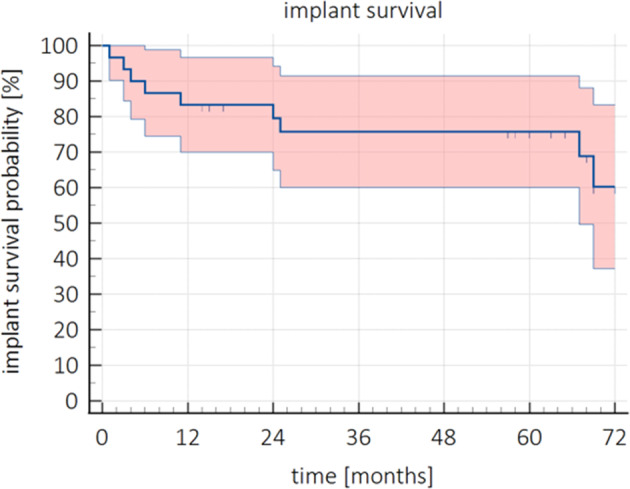
Survival probability (%) over time (months) with 95% CI of the ZV3 implants.

Survival probability of the ZV3 implant was determined by the Kaplan–Meier analysis with 95% CI as shown in Figure 5. In total, nine out of 30 implants were lost. Causes were early implant failure (*n* = 4) and mechanical failure (*n* = 5). Additionally, three subjects were lost to follow‐up (patients who died during the course of the study (*n* = 2), and the patient who could not be reached (*n* = 1)). Median time of follow‐up was 63.0 (IQR = [16.0–69.0]) months. Survival probability of the ZV3 implant after 60 months was 75.8% (95% CI [60.0%; 91.0%]) and 59.6% (95% CI [49.4%; 69.9%]) at the end of the study (77 months).

**FIGURE 6:**
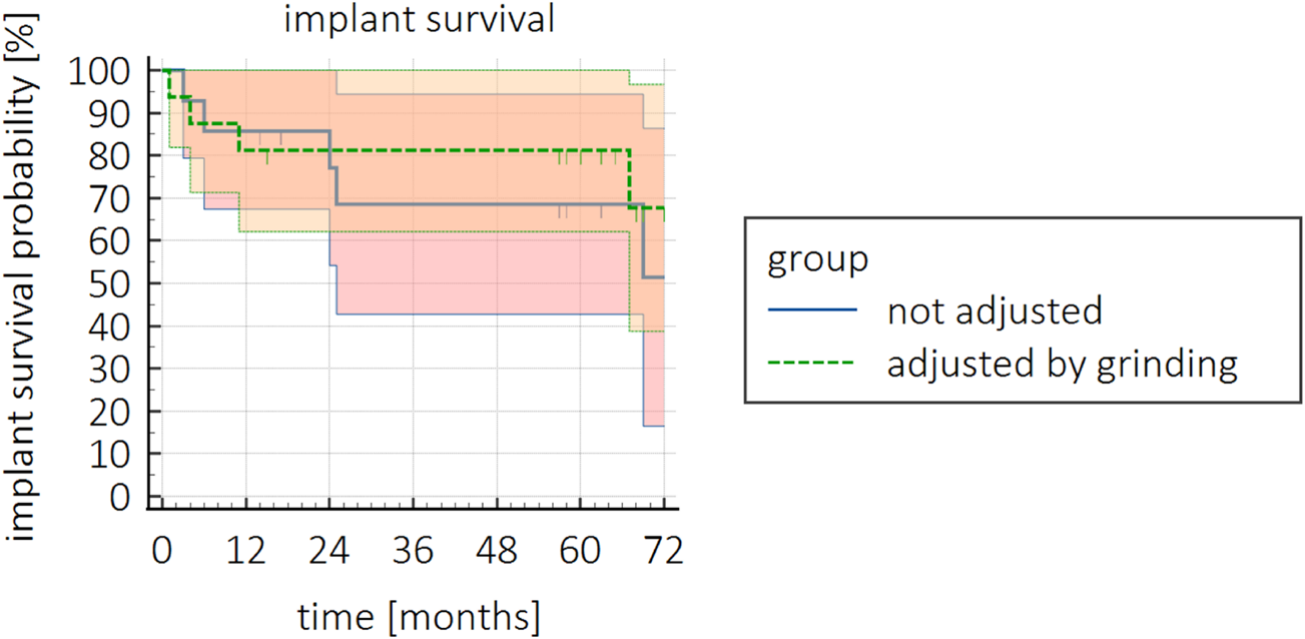
Survival probability (%) over time (months) with 95% CI of the ZV3 implants stratified for manipulated or not manipulated implants.

To determine the influence of the implant been manipulated buccally on the survival of the ZV3 implant, the Kaplan–Meier survival analysis was also performed with stratification by manipulated or not manipulated implants. The survival probability of an implant at T60 was 81.3% in the manipulated implants group and 68.6% in the not manipulated group. The log‐rank test showed that the difference between the two groups was not significant at the end of the study period (*χ* ^2^ (1) = 0.290; *p* = 0.590). Survival by means of these criteria is presented in Figure 6.

**FIGURE 7:**
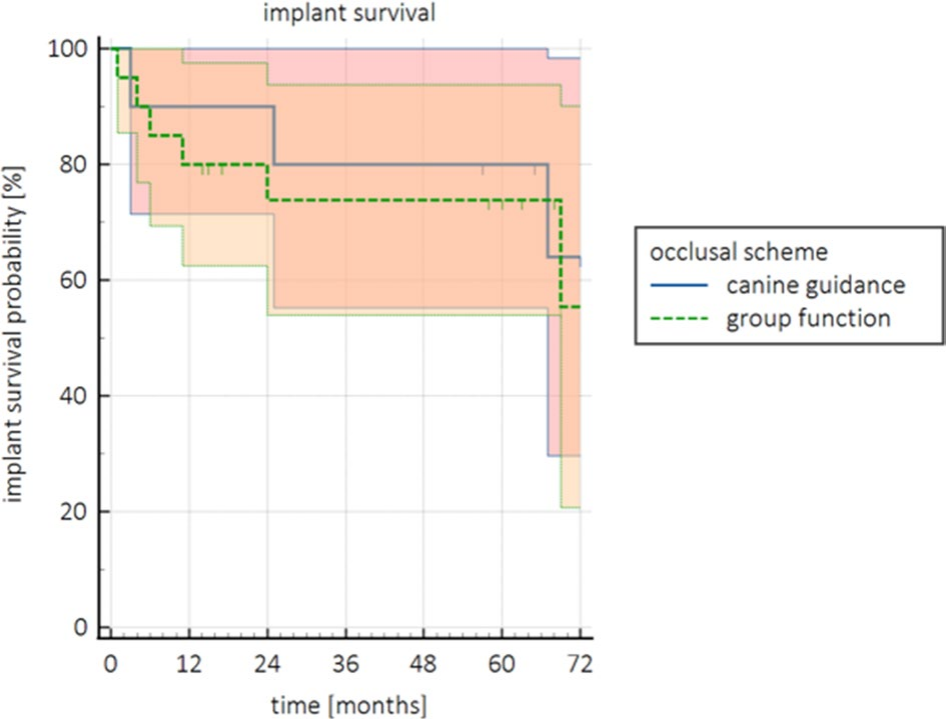
Survival probability (%) over time (months) with 95% CI of the ZV3 implants stratified for two groups of occlusal schemes: canine guidance and group function.

To determine the influence of the kind of occlusal scheme of the subject on the survival of the ZV3 implant, the Kaplan–Meier survival analysis was performed with stratification by means of the two defined groups. The estimated survival probability of an implant at T60 was 80.0% in the group patients with canine guidance and 73.8% in the group patients with group function. The log‐rank test showed no significant differences between the two groups at the end of the study period (*χ* ^2^ (1) =0.106; *p* = 0.745). Survival curves by means of these criteria are shown in Figure 7.

### 3.3 Implant Success

**FIGURE 8:**
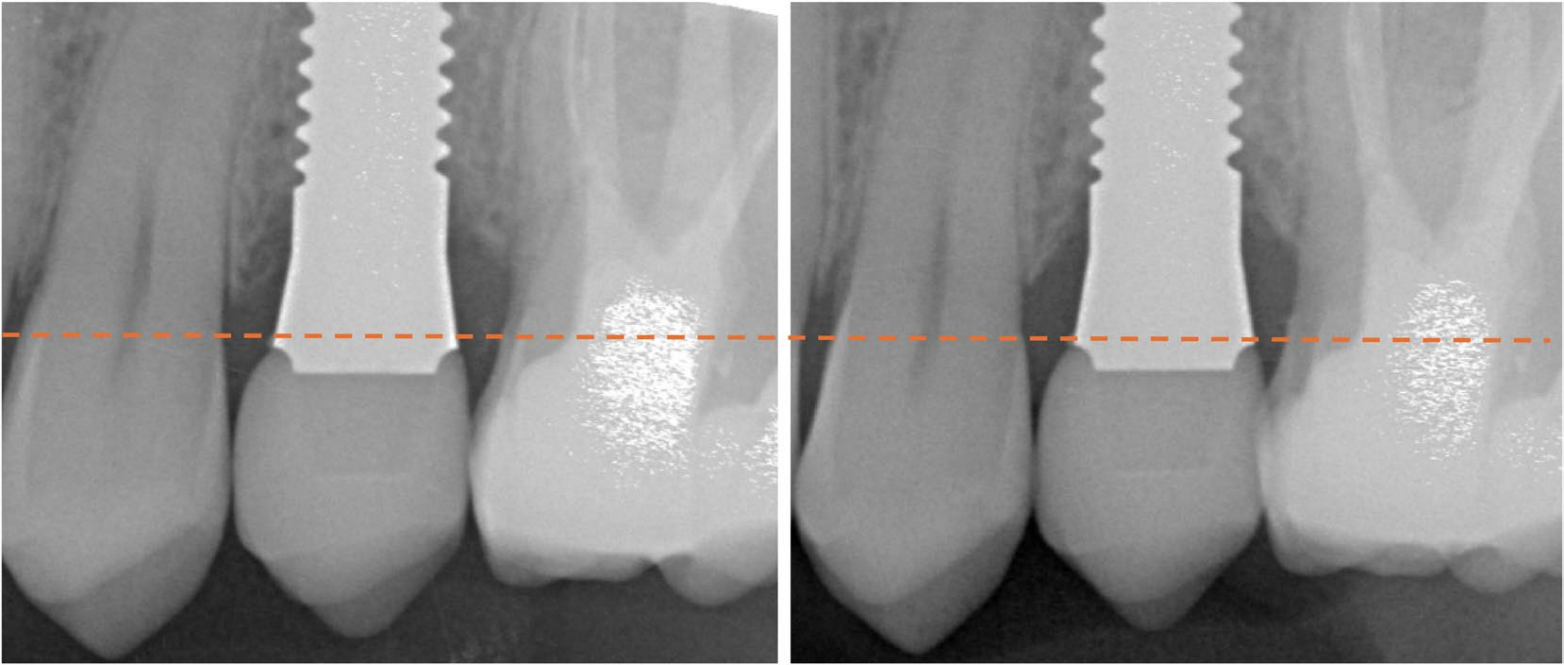
Radiographic image of the implant (a) 1 month (T1) and (b) approximately 5 years (T60) after crown placement with reference line for the marginal bone‐level measurement on the calibrated intraoral radiographs.

Possible peri‐implant radiolucencies and MBLs were examined on a radiograph (Figure 8). Peri‐implant radiolucencies were not identified. Mean (most severe) MBL change between baseline and first follow‐up after 1 year was −0.26 mm (minimum −0.99 mm, maximum 0.91 mm, SD = 0.45 mm) and between baseline and second follow‐up after 5 years was −0.25 mm (minimum −0.91 mm, maximum 1.10 mm; SD = 0.54 mm). Peri‐implant bone loss over 0.2 mm annually after the first year after implant service was not detected. No statistically significant differences in MBL between the three time points could be found among surviving implants, with *p* = 1.00 for all comparisons.

**FIGURE 9:**
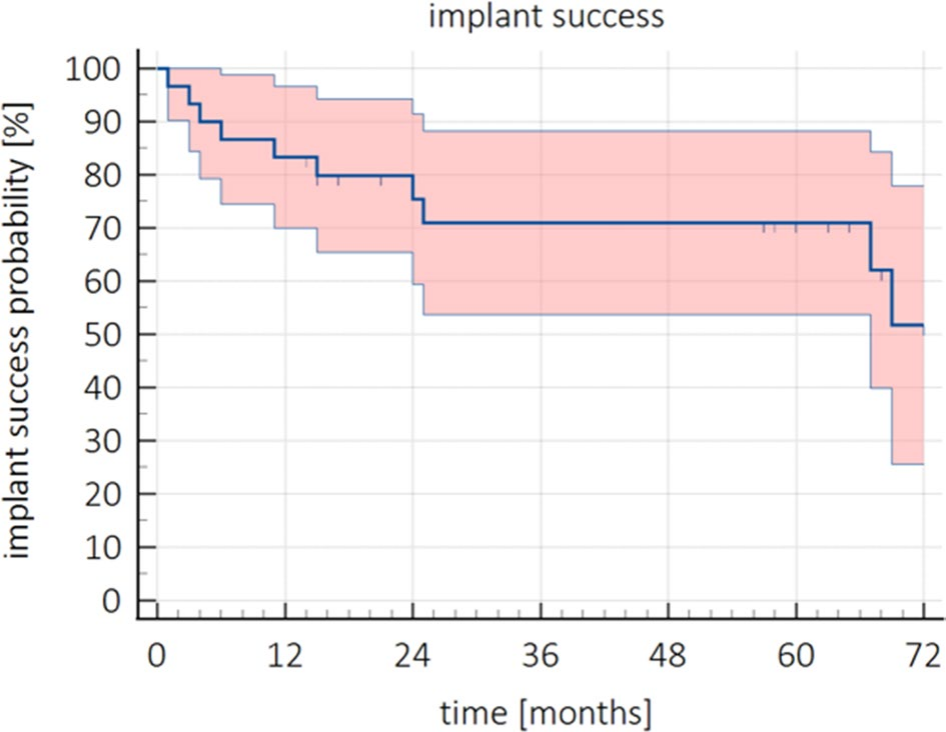
Success probability (%) over time (months) with 95% CI of the ZV3 implants.

Scores of the clinical parameters were generally favourable on T1, T12, and T60 (Table 3), with median scores of 0 for all parameters, indicating generally healthy peri‐implant tissues. However, at T60, one heavily bleeding site was found in one subject, which was then categorized in the peri‐implant mucositis group and so as not being a success (*n* = 1). In total, 9 out of 30 implants failed and thus not a success (*n* = 9). In total, 10 implants were categorized as not successful, and six subjects were lost to follow‐up to determine success at T60. Median time of follow‐up was 55.0 (IQR = [15.0–65.8]) months. In Figure 9, the Kaplan–Meier success curve with 95% CI for the ZV3 implants is given. The probability of success of the ZV3 implant after 60 months was 71.0% (95% CI [54.0%; 88.0%]).

**TABLE 3:** Mean clinical outcome measures of the implant site at T1 (baseline), T12 (1 year), and T60 (5 years).

	Baseline (T1) (*n* = 27)	T12 (*n* = 25)	T60 (*n* = 14)
Plaque index			
Score 0	77.8%	76.0%	64.3%
Score 1	22.2%	24.0%	35.7%
Score 2			
Calculus index			
Score 0	100.0%	100%	85.7%
Score 1			14.3%
Bleeding index			
Score 0	59.3%	72.0%	85.7%
Score 1	33.3%	16.0%	7.1%
Score 2	7.4%	8.0%	7.1%
Score 3		4.0%	
Mean probing depth, mm (SD)	2.4 (0.75)	2.6 (1.0)	2.7 (1.2)

**FIGURE 10:**
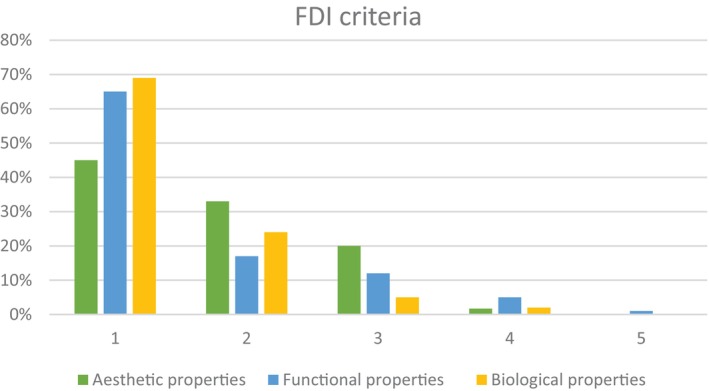
Scores in the categories of the modified FDI criteria—aesthetic criteria, functional criteria, and biological criteria. Scores for the implant‐supported crowns assessed 60 months (T60) after crown placement. Five scoring‐categories can be distinguished in the modified FDI clinical criteria: score 1 = clinically excellent/very good, score 2 = clinically good, score 3 = clinically sufficient/satisfactory, score 4 = clinically unsatisfactory, score 5 = clinically poor [48]. *p values < 0.05 were considered significant.

Of the 30 placed implants, 28 received a crown. Two implants showed early osseointegration failure after crown placement (*n* = 2) and five implants fractured (*n* = 5). Two patients died (*n* = 2) and five patients were lost to follow‐up (*n* = 5). The remaining implant‐supported crowns (*n* = 14) were qualitatively evaluated using the FDI criteria. The aesthetic, functional, and biological scores are presented in Figure 10. No interventions were required, and no complications were described or mentioned by the participants with surviving implants. Scores of the occlusal contour and wear deteriorated over time but remained clinically sufficient. The strength of the mesial and distal contact points, within criteria approximal form, decreased over time.

### 3.4 Patient‐Reported Outcomes

**FIGURE 11:**
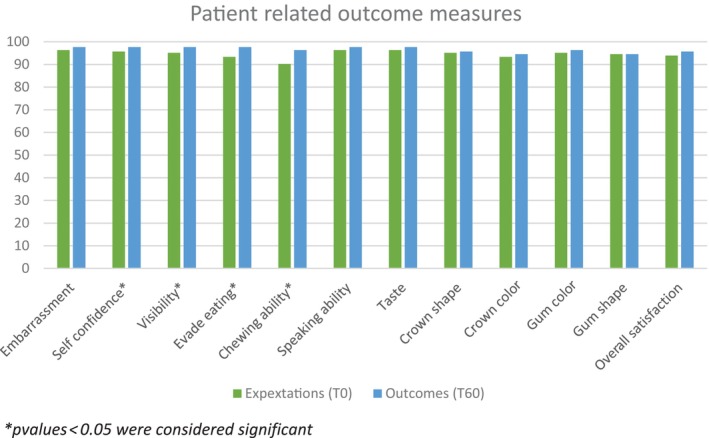
Patients' expectation (T0) and satisfaction outcomes at second follow‐up (T60) for 12 statements. *p‐*values displayed for the comparison across time using Wilcoxon signed‐rank tests between patients' expectations (T0) and patients' satisfaction at second follow‐up (T60).

Median expected overall satisfaction preoperative was 93.9% (IQR = [86.9%–96.3%]) and median overall satisfaction at the second follow‐up was 95.7% (IQR = [91.5%–97.6%]). Patients experienced significantly more self‐confidence (*p* = 0.035), confidence in loading of the implant (*p* = 0.004), a better chewing ability (*p* = 0.005), less visibility of the implant (*p* = 0.006), and less pain during function (*p* = 0.005) than they had expected before treatment. Overall scores were generally high and expectations were met (Figure 11).

## 4 Discussion

The aim of this prospective 5‐year follow‐up single‐arm clinical trial was to evaluate the use of custom‐made two‐piece metal‐free ZV3 implants by establishing the survival and success rate of the implants and the implant‐supported crown, various clinical parameters, and PROMs.

The used two‐piece ZV3 implants are customized per patient: both the body of the implant and the emergence profile were individually designed to fit the available bone volume and support the soft tissues. Brüll et al. [39] suggested a favorable bending strength, fracture toughness, and Weibull modulus on the same custom‐made (one and two‐piece) ZV3 implants as used in the present study. However, the final mechanical strength of an individual implant depends on both the manufacturing process as well as the final design [23]. This could potentially be an issue when manufacturing a custom‐made implant. In the literature, very little has been reported on custom‐made ZrO_2_ implants. Also, for two‐piece ZrO_2_ implants, mid‐ or long‐term literature is rare [26].

Survival and success rates were calculated to evaluate the clinical performance of the ZV3 implants. Implant survival probability in this study was 83.3% (95% CI [70%; 97%]) and 75.8% (95% CI [60.0%; 91.0%]) after 1 and 5 years, respectively. This is considered poor when compared to the results found in a review of zirconia implants [16], where a survival rate of 95.6% after 1 year is reported. One study reported on an 85.7% survival rate after 80 months for two‐piece ZrO_2_ implants [37]. Both survival rates are higher than the survival probability of the implants in this study. After the first year, a calculated expected loss in the survival rate of 0.05% annually was expected in the previous study by Pieralli et al. [16], which is not in line with the current study. Also, most implant failures in the same study were reported before prosthetic loading (failed osseointegration), whereas in the present study, half of the failed implants were lost after prosthetic loading. Altogether, implant loss in this study was higher than anticipated.

Horizontal fracture of the ZrO_2_ implant was seen in five patients. The implant could be removed surgically in three cases. In the other two cases, the remaining part remained in the maxilla. Mechanical implant failures constitute great discomfort for the patients. Hence, reimplantation or other restorative solutions had to be found. Reasons for titanium implant fractures are described in the literature [49]. Possible risk indicators for early and late implant loss [49] were not taken into account in the current study. Pieralli et al. [16] reported most implant failures in the early period after placement. Padhye et al. [6] concluded a higher number of early implant failures within 1 year after loading compared to titanium implants. Both were seen in this study. However, no recent literature reported on the high rates of implant fracture as reported in the current study.

One of the intentions of this single‐arm trial was to demonstrate the clinical performance within our clinic, before offering ZV3 ceramic implants to the regular patient. Therefore, we adapted the “Therapy Guide for ZV3 Ceramic Implants” to our clinical needs. Among other recommendations, it was suggested to “position the implant in the crown axis, not off‐set, if not possible, augment by, for example, bone splitting.” The implants in this study were placed in the native bone rather than performing bone augmentation. Another recommendation was to “avoid cuspid contact during lateral movements.” We complied but established steep cusp angulation where aesthetically needed, as we would have done in titanium implant placement. It seems possible that antagonistic teeth elongated after placement of the implant crown and established high lateral forces during dynamic occlusion.

Both factors might have played a role in the mechanical overloading of the implants [41], whereas other recommendations that were not followed like monitoring the vitamin D blood value, ozonizing the prepared bone, platelet‐rich plasma treatment, or testing osseointegration with a Perio tester might have been of minor importance.

Becker et al. and Brunello et al. [50, 51] reported on the same cohort of patients and used ZV3 standard implants in a prospective study. It is noteworthy that of 30 patients who attended the long‐term follow‐up (up to 9 years) one decementation and six fractures of the fiberglass post occurred, whereas no fracture of the implants was observed, neither during the short‐term (2 years) or long‐term follow‐up period. In contrast, in the present study, no prosthetic problems were encountered, whereas five horizontal implant fractures occurred. Inclusion criteria, as well as prosthetic protocol, location of the implants of interest, or palatal placement of the implants of the present study have been intensely discussed with the researchers of both groups, without being able to identify one of those variables as a main contributor to the different appearances of failure. Also, Brüll et al. [39] reported in a retrospective evaluation on 121 ZV3 implants, of which 66 the same two‐piece design as used in the current study. They reported an estimated survival rate of 96.5% after 3 years, comparable to those reported in the literature [52].

Most studies in implant dentistry describe titanium implants [52, 53]. Mentioned rates of implant fracture with titanium implants of 0.5% after 5 years, which is much lower than in the current study. One study of another brand of ZrO_2_ implants showed a fracture rate of approximately 8% after 3 years after prosthetic loading [22]. The fractured implants had been located in maxillary and mandibular anterior regions. Presumed causes of all mechanical implant fractures was overload. This is worrying because occlusal forces in the anterior region tend to be approximately three times lower than occlusal forces in posterior regions [54]. According to a review about fracture resistance of ZrO_2_ implants, two‐piece implants are even less fracture‐resistant than one‐piece implants [23]. In addition, it was concluded that manipulation of the ZrO_2_ implant should be avoided due to a negative impact on fracture resistance. Gahlert et al. [22] mentioned little damages resulting from post sintering roughening of the surface of a ZrO_2_ implant as a possible reason for local material stress which could initiate cracks. Regarding the survival rates of the manipulated implants and of mechanical overloaded implants due to the kind of occlusal scheme in this study, meaningful comparisons with other literature were ruled out due to the use of different criteria.

The survival probability of an implant after 5 years was 81.3% in the manipulated implants group and 68.6% in the non‐manipulated group, but this difference did not reach a statistically significant level and as a consequence is not in line with the aforementioned literature. It can be suggested that those differences are most likely based on random effects in the distribution, but manipulating the ZV3 implants does not seem to affect the survival negatively. The estimated survival probability of implants at T60 was 80.0% in the group patients with canine guidance, and 73.8% in the group patients with group function [55]. Emphasized the importance of decreased cusp inclination in lateral excursion in combination with disclusion of the posterior region to protect an implant. Although legitimate, this suggestion could not be substantiated by the results of the present study.

In addition to implant survival, ZV3 ZrO_2_ implant success was determined using the criteria of success as described by Albrektsson et al. [42]. The implant success rate of 71.0% (95% CI [54.0%; 88.0%]) after 5 years in the current study was lower than reported in the literature. According to Kohal et al. [56] peri‐implant bone‐level evaluation is a pivotal criterium in determining the success of a dental implant. Hence, peri‐implant bone alteration was evaluated after approximately 1 and 5 years in this study. Mean (most severe) MBL change between baseline and first follow‐up after 1 year was −0.26 mm (minimum −0.99 mm, maximum 0.91 mm, SD = 0.45 mm) and between baseline and second follow‐up after 5 years was −0.25 mm (minimum −0.91 mm, maximum 1.10 mm; SD = 0.54 mm). No significant average MBL changes occurred between 1 and 5 years. Peri‐implant bone alteration at the surviving implants was comparable with reported in other studies regarding ZrO_2_ implants in the literature [26].

No complications were seen regarding the implant‐supported crowns. The evaluation of the implant‐supported crowns was determined with help of the FDI World Dental Federation clinical criteria [48]. Within the aesthetic criteria, it was seen that surface lustre, colour match, and translucency deteriorated over time. A study, in which a different type of lithium disilicate restorations was analyzed, also showed deteriorating results for surface lustre, colour match, and translucency [57]. As possible explanation of worsening of the lustre, the loss of glaze was given. In addition, a recent study [58] showed lithium disilicate restorations after 1 year that lost glaze and showed a modification in colour and translucency. Over time, the colour of natural teeth may darken, staining on the teeth, recessions of the gingiva, or a combination of factors may occur. It is plausible that the crown material and natural dentition do not age in the same way, which may also be a factor to consider contributing in the difference in colour match over time.

Concerning the functional properties criteria, it is particularly noticeable that a total of nine implant‐supported crowns lacked a strong contact point over time. In comparison with other studies, which also used a 50 μm strip for measurements, that reported an approximal contact point loss of 43% [59] and 58% [60], the rate of 30% in this study was relatively low and comparable to the literature [61]. In this study, the approximal contact points were evaluated with metal strips of three different thicknesses (25, 50, and 100 μm) determine whether they were strong enough.

Satisfaction with regard to all statements was higher than patients' expectations at baseline, first follow‐up, and second follow‐up. Furthermore, satisfaction was consistently high over the follow‐ups, which indicates a general contentment with the treatment result. Patients experienced significantly more self‐confidence, confidence in loading of the implant, a better chewing ability, less visibility of the implant, and less pain during function than was expected. Patients' satisfaction was particularly high shortly after crown placement. However, satisfaction gradually decreased over time following baseline measurements after crown placement. Presumingly, a positive feeling of receiving the crown might have affected the satisfaction measurement shortly after, rather than the satisfaction actually decreasing over time.

One periodontal failure was seen with regard to the biological criteria. The adjacent mucosa reacted with heavy bleeding after probing as a result of poor oral hygiene during the follow‐up period. The periodontal response is unlikely to directly affect the implant‐supported crown but rather affects the environment around the implant and its survival.

A limitation of the findings in this prospective 5‐year follow‐up single‐arm clinical trial is the low number of subjects. However, the main focus in this study was to evaluate this particular custom‐made two‐piece ZrO_2_ dental implant as a reliable treatment option in clinical practice. The small sample and the relatively high number of no‐shows affected the reliability of the statistical analyses. This resulted in large confidence intervals in the survival and success analyses. Hence, the survival probability after 5 years should be interpreted with caution. Additionally, small differences over time may be unnoticed due to the small number of patients. The current single‐arm study design does not provide the opportunity to demonstrate the benefit of this treatment. Comparison with other data is necessary; however, this has its limitations due to the choice of references and other biases as difference in measurements and process within other study settings [62].

## 5 Conclusion

Results show a survival probability after 5 years of 75.8% (95% CI [60.0%; 91.0%]) for the ZV3 implant. Survival rate of the ZV3 implants that were used in this study is lower than expected. Based on these findings, the indication for ZV3 ZrO_2_ implants as applied in this study cannot be recommended for the clinical practice. Lateral forces should be taken into account when off‐axis placement of the ceramic implant is performed. Further research on the different appearances of mechanical failure in ZrO_2_ implants is highly recommended.

## Author Contributions

J.H.W.B. data analysis/interpretation, drafting article, critical revision of article, statistics, data collection. M.S.C. concept/design, drafting article, critical revision of article, approval of article, funding. H.J.A.M. and G.M.R. concept/design, critical revision of article, approval of article, acquisition. U.S. concept/design, data analysis/interpretation, drafting article, critical revision of article, data collection, funding.

## Conflicts of Interest

The authors declare no conflicts of interest.

## Data Availability

The data that support the findings of this study are available from the corresponding author upon reasonable request.
